# Optimizing Screening for Early Disease Detection in Familial Pulmonary Fibrosis (FLORIS): A Prospective Cohort Study Design

**DOI:** 10.3390/jcm12020674

**Published:** 2023-01-14

**Authors:** Martijn T. K. Maus, Karlijn Groen, Joanne J. van der Vis, Jan C. Grutters, Coline H. M. van Moorsel

**Affiliations:** 1 St. Antonius ILD Center of Excellence, Department of Pulmonology, St. Antonius Hospital, Koekoekslaan 1, 3435 CM Nieuwegein, The Netherlands; 2 Department of Clinical Chemistry, St. Antonius Hospital, Koekoekslaan 1, 3435 CM Nieuwegein, The Netherlands; 3 Division of Heart and Lungs, University Medical Center Utrecht, 3584 CX Utrecht, The Netherlands

**Keywords:** screening, early detection, familial pulmonary fibrosis, interstitial lung disease, interstitial lung abnormalities

## Abstract

**Background**: Familial pulmonary fibrosis (FPF) can be defined as pulmonary fibrosis in two or more first-degree family members. The first-degree family members of FPF patients are at high risk of developing FPF and are eligible for screening. Reproducible studies investigating risk factors for disease are much needed. **Methods**: Description of the screening study protocol for a single-center, prospective cohort study; the study will include 200 asymptomatic, first-degree family members of patients with FPF who will undergo three study visits in two years. The primary objective is determining the diagnostic value of parameters for detection of early FPF; the secondary objectives are determining the optimal timing of the screening interval and gaining insight into the natural history of early FPF. The presence of interstitial lung disease (ILD) changes on high-resolution computed tomography of the chest is indicative of preclinical ILD; the changes are determined at baseline. The comparison between the group with and without ILD changes is made for clinical parameters (pulmonary function, presence of digital clubbing, presence of Velcro-like crackles, blood count, liver- and kidney-function testing, patient-reported cough and dyspnea score) and exploratory parameters. **Discussion**: This study will be the first large-size, prospective, longitudinal cohort study for yearly screening of asymptomatic family members of FPF patients investigating the diagnostic value of parameters, including lung function, to detect early FPF. More effective screening strategies could advance early disease detection.

## 1. Introduction

Within the group of interstitial lung diseases (ILD), pulmonary fibrosis (PF) is a chronic, often progressive process associated with high mortality. Studies show that 5–25% of patients with PF have familial disease [1,2,3,4]. Although definitions vary between studies, here we define familial pulmonary fibrosis (FPF) as PF being present in two or more first-degree family members. FPF most commonly presents as idiopathic pulmonary fibrosis (IPF) [2,3,4], but other ILDs such as rheumatoid arthritis (RA)-ILD, hypersensitivity pneumonitis (HP)-ILD and unclassifiable ILD are also observed [1,2].

FPF is usually inherited in an autosomal-dominant way. In that case, first-degree relatives of FPF patients have a 50% chance of also carrying the disease-causing mutation and are therefore at high risk of developing FPF [3,4,5]. Disease course, including decline in forced vital capacity (FVC) [6] and mortality [2,4,7,8] in FPF patients, is comparable with sporadic IPF. While there is no specific treatment for adult FPF patients, retrospective analyses suggest that antifibrotic treatment has similar safety and effectivity as in patients with IPF or progressive PF [9,10,11]. In studies of patients with FPF, diffusing capacity for carbon monoxide (DLCO) and FVC are often already impaired at diagnosis, with values around 50% and 70% of normal, respectively [6,12,13]. Timely diagnosis is thus important to fully benefit from therapeutic intervention.

Current guidelines of the Dutch Association of Clinical Genetics recommend actively informing first-degree relatives of patients with genetic diseases about their increased risk for disease and the possibilities of genetic screening [14]. The St Antonius ILD Center of Excellence and the Universitair Medisch Centrum (UMC) Utrecht Clinical Genetics Department have put in place a clinical protocol to screen for early pulmonary disease in asymptomatic, high-risk relatives older than 18 years of age. Current clinical screening involves a high-resolution computed tomography (HRCT) of the chest with a five-year interval and an annual medical examination consisting of anamnesis, pulmonary function test, blood tests, and physical examination. This screening protocol was based on parameters that are used for the detection of PF in symptomatic persons, as well as information gathered from studies investigating patients with FPF or asymptomatic family members [6,15,16,17]. However, evidence on the diagnostic value of these markers, notably in early disease, is scarce and the intervals were chosen arbitrarily.

The value of an HRCT scan as a screening tool in asymptomatic, at-risk relatives is evident. Several studies have investigated the prevalence of ILD changes on HRCT scans in asymptomatic relatives of familial or sporadic PF patients. They reported surprisingly congruent percentages of 14–23% of relatives with ILD changes [15,17,18,19,20]. However, data for other potentially interesting screening parameters, including pulmonary function, are limited. As yearly radiographic imaging in healthy subjects is not advised due to radiation exposure and costs, other screening parameters to reliably detect disease development and progression are needed. Additionally, little is known about the early course of disease in FPF, and data from screening and follow-up programs will be most informative.

In this article, we describe the design of the FLORIS study (optimizing screening detection for early disease detection in FamiLial pulmOnaRy fibrosIS) which will include 200 first-degree family members of patients with FPF and is currently in progress. The presence of ILD changes on the baseline HRCT scan will be used as the indicator for presence of early pulmonary disease, and we will assess the predictive value and follow-up dynamics of lung function, physical examination, blood markers, and questionnaires. In addition, the aim is to provide insight into the natural history of early familial disease as this is currently largely unknown.

## 2. Materials and Methods

### 2.1. Objectives and Endpoints

**Figure 1:**
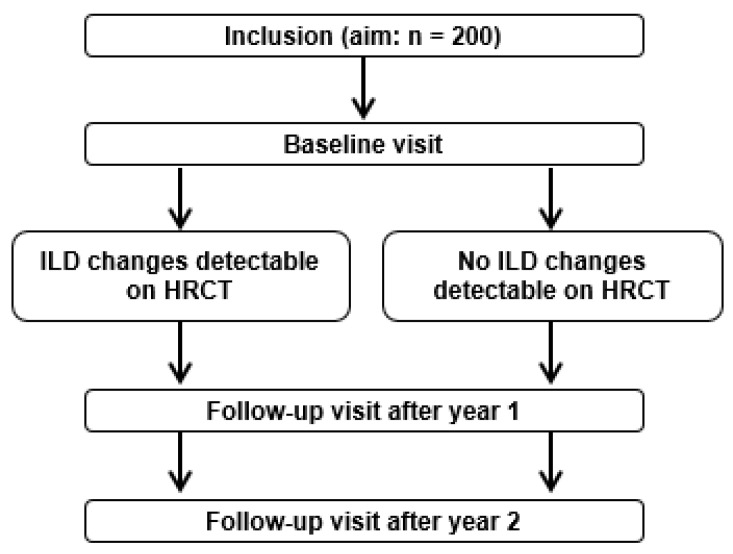
Study groups of the FLORIS study. Definition of abbreviations: ILD = interstitial lung disease and HRCT = high-resolution computed tomography.

The primary objective of the FLORIS study is to determine the diagnostic value of parameters for early disease detection in first-degree relatives of patients with FPF. To this end, the presence or absence of ILD changes on a baseline HRCT scan (see Figure 1) will be used to determine the predictive value for early disease of a set of clinical and exploratory parameters (see Table 1).

As secondary objectives, the study aims to gain insight into the natural history of FPF and to determine the necessary interval between screening visits. Secondary endpoints will assess the change in parameters between baseline and follow-up after one and two years within each group separately, and analyze differences between the groups with and without ILD-changes (see Figure 1).

### 2.2. Design and Participants

The FLORIS study is a prospective, single-center study performed at the ILD Center of Excellence at the St Antonius Hospital in Nieuwegein, The Netherlands. Two hundred first-degree family members of patients with FPF will be screened according to a predefined screening protocol, with a two-year follow-up. Participants must be first-degree relatives of patients with FPF, asymptomatic, and at least 18 years old. Exclusion criteria are a previous diagnosis of ILD or pregnancy at baseline. If a participant becomes pregnant during the follow-up, they will not be excluded. Potential participants will receive study information via clinical geneticists of the UMC Utrecht, through the national ILD section of the Dutch Society of Pulmonologists, and through other sources such as the PF patient foundation. Current patients with FPF will also be given a letter with information about the study which they can hand to their family members. If patients are willing to participate, written informed consent will be obtained, and patients will be screened for eligibility.

### 2.3. Hospital Visits

Hospital visits will take place at baseline, after 1 year (±1.5 months) and after 2 years (±1.5 months). Study procedures include the standard of care procedures as present in the current clinical screening protocol. Additionally, several exploratory parameters are investigated (see Table 1). At baseline, patient characteristics including age, sex, smoking status, family health history and ILD-related history will be collected and an HRCT scan will be performed to assess the presence of ILD changes. During each visit, participants will be examined by a physician. Pulmonary-function tests will also be performed, blood samples will be collected and patients will fill out digital questionnaires. Study measurements are described in Table 1. Any health event occurring to the participants during the study will be noted upon each study visit.

### 2.4. Assessment of Preclinical ILD

At baseline, subjects will be stratified into two groups based on the presence (ILD changes group) or absence (no ILD changes group) of ILD changes on HRCT scan (Figure 1). ILD changes are defined as extensive disease, familial interstitial lung abnormalities (FILA) or minimal FILA (see Box 1). The definition of FILA is based on the position paper of the Fleischner Society on interstitial lung abnormalities (ILA) [21]. The position paper does not consider abnormalities identified during screening for ILD in high-risk groups, including familial ILD, as ILAs because they are not incidental. The Society suggests the term preclinical ILD for such cases, however, this suggests that subjects have ILD which is not always the case. Therefore, we added familial (F) to refer to the context of the findings, which together makes FILA. To define all possible abnormalities during screening, minimal FILA will be defined as FILA in less than 5% of the lung zone (see Box 1).

A thoracic radiologist will assess the HRCT scan. All the findings of study visits will be discussed with an expert pulmonologist in the field and, when necessary, in a multidisciplinary discussion. If ILD changes or other abnormalities in clinical parameters are found that warrant evaluation, participants will remain in the study, but will also be referred to regular care for examination by a general practitioner or pulmonologist.

Box 1 **Definition of ILD changes in the FLORIS study.**

ILD changes on HRCT scan in first-degree family members of FPF patients are defined as:

- Extensive disease: non-trivial abnormalities present in three or more lung zones (a)
- FILA: Any of the following non-dependent abnormalities involving >5% of a lung zone: traction bronchiectasis, honeycombing, ground-glass or reticular abnormal-ities, lung distortion and non-emphysematous cysts (a)
- Minimal FILA: Any of the following non-dependent abnormalities involving <5% of a lung zone: traction bronchiectasis, honeycombing, ground-glass or reticular abnormalities, lung distortion and non-emphysematous cysts

(a) Concepts are based on the definition of ILA by Hatabu et al. [21] for incidental findings and adjusted to a screening setting of high-risk relatives

### 2.5. Parameters

#### Physical Examination

At each study visit, a physician will inspect the presence of digital clubbing and perform lung auscultation. Bilateral clubbing is a common phenomenon in ILD (e.g., IPF, HP, sarcoidosis) [22]. In familial IPF, finger clubbing was seen in 13–30% of the patients at diagnosis [6,7].

Studying auscultation, Moran-Mendoza et al. observed that fine crackles were present in 93% of IPF patients upon initial presentation [23]. Similar findings are found in familial IPF patients, with studies reporting Velcro-like crackles at diagnosis in 93–96% of the patients [6,7]. The presence of Velcro-like crackles may hence be a valuable tool in early diagnosis. A recent review on early diagnosis of fibrotic ILD mentioned the training of primary care physicians for better identification of Velcro-like crackles and electronic auscultation tools, as possible aids in the process of auscultation during the diagnostic progress [24]. In RA patients, analysis for Velcro-like crackles by an algorithm of recordings of an electronic stethoscope showed a high diagnostic accuracy for ILD, with a sensitivity of 93.2% and a specificity of 76.9% when compared to HRCT scan [25].

In the FLORIS study, participants will undergo digital auscultation in addition to the standard analogue method with a non-validated, electronic stethoscope (eKuore One Wireless, Valencia, Spain). This otherwise regular stethoscope is connected to a device that records lung sounds, which can then be transferred through Bluetooth to a smartphone application. This allows the sound recordings to be linked to the exact position of the lung. Afterwards, computer analysis will be performed for the detection of Velcro-like crackles.

### 2.6. Lung Function Testing

Lung function tests consist of spirometry, single-breath diffusion and body box. Key variables are FVC, DLCO (corrected for hemoglobin) and total lung capacity (TLC). DLCO is measured by the standard diffusion test, i.e., the carbon-monoxide diffusing lung capacity single-breath technique. TLC is measured in the body box. In relatives of patients with PF, those with ILA showed relative reductions in FVC, TLC and DLCO [16].

### 2.7. Blood Markers

Blood samples will be taken at every visit to measure the standard of care, complete blood count and liver and kidney function tests. This is of particular interest as mutations in telomere-related genes cause a significant proportion of FPF. Hematological abnormalities are rather common in patients with telomere-related disease, with red blood cell macrocytosis in 24–41%, thrombocytopenia in 8–54% and anemia in 17–27% of patients [12,13,26,27]. In individuals with telomere-related gene mutations, liver disease is also seen, and liver enzymes can be elevated [28,29]. In screening of at-risk subjects, liver disease was reported in around 2.7–5% of subjects [15,17]. Kidney disease has also been linked to short telomere syndrome [30]. In the exploratory segment of the study, the levels of the blood biomarkers Krebs von den lungen 6 (KL6), surfactant protein-D (SP-D), Chemokine (C-C motif) ligand 18 (CCL18) and matrix metalloproteinase 7 (MMP7) will be analyzed. Elevated levels of KL-6, SP-D and MMP7 were associated with a diagnosis of PF according to a meta-analysis [31]. Interestingly, Kropski et al. found that increased levels of MMP7 and SP-D were significant predictors of HRCT scan abnormalities in asymptomatic family members of patients with familial disease when compared with healthy control subjects [17]. Moreover, elevated levels of KL6 [32], CCL18 [33] and SP-D [34,35] have been associated with lung function decline or poor survival in IPF. Biomarker analysis will be performed according to the standard protocol in our laboratory [36,37]. Samples may be used to explore other putative blood biomarkers in addition to the aforementioned known biomarkers.

### 2.8. Questionnaires

Subjects will receive digital questionnaires prior to each visit (see Table 1). Hunninghake et al. did not observe a significant difference in the frequency of reported respiratory symptoms between relatives with or without ILA screened for PF [16]. On the other hand, a retrospective study by Hewson et al. showed that IPF patients were significantly more likely to present with symptoms of dyspnea and cough, and to a lesser extent fatigue, in the five years prior to diagnosis [38]. Patient-reported parameters on respiratory symptoms may thus be potentially informative for early disease detection.

The Medical Research Council (MRC) dyspnea scale was assessed longitudinally in chronic ILD. A MRC dyspnea score at baseline (in IPF) or an increase in score over time (in IPF and non-IPF ILD) has been associated with clinical progression of the disease [39]. Cough is a major symptom in IPF [40], and an independent predictor of disease progression in IPF [41]. Self-reported cough severity can be measured through a visual analogue scale (VAS) [42]. It is composed of a 100 mm scale with the extremes at ‘no cough’ and ‘worst possible cough severity’, whereby the subjects mark their cough severity.

In order to better understand the natural history of PF, two additional questionnaires on fatigue and health status will be administered to investigate whether symptoms not directly related to the lung may be present. The Fatigue Assessment Scale (FAS), a 10-item general fatigue questionnaire, will be used to measure fatigue. To measure health status, the EuroQOL-5 Dimensions (EQ-5D) will be used. This tool consists of two parts: a 5-dimension list (mobility, self-care, usual activities, pain/discomfort and anxiety/depression) with a 5-level classification (ranging from no problems to extreme problems), and the EQ-VAS. Both the FAS [43] and the EQ-5D [44,45,46] have been used in ILD before.

Other questionnaires at baseline will include ‘demographics and expositions’ and ‘diseases in the family’, which are already standard procedure at the ILD outpatient clinic; there will be a short follow-up questionnaire at visits 2 and 3. A non-validated questionnaire with questions regarding risk perception, reasons for participating and prior screening will also be included at baseline.

### 2.9. MUC5B rs35705950 Genotyping

The minor allele of the *MUC5B* promoter variant rs35705950 is an important risk factor for IPF and FPF, with a minor allele frequency of over 33% in white patients in contrast to 9% in healthy controls [47]. Recent studies showed that this *MUC5B* risk allele is also associated with other forms of PF [48,49,50,51,52]. Amongst asymptomatic relatives, signs of preclinical PF on HRCT scan were significantly, although only in relatives aged over 60 years [19] or borderline significantly [15] more common in carriers of the *MUC5B* risk allele. Genotyping will be performed according to the protocol described in Van Der Vis et al. 2016 [50].

### 2.10. Six-Minute Walk Test

The six-minute walk test (6MWT) is used in patients with chronic respiratory disease, with the test being primarily a test of physical capacity [53,54]. Walking distance, heart rate and O2 saturation (sat), together with subject length, weight, Borg Dyspnea and Leg Fatigue scales (1–10), will be noted according to standard protocol. Previous studies showed a shorter six-minute walk distance is present in patients with an increased risk of mortality for patients with IPF, chronic obstructive pulmonary disease, ILD, pulmonary hypertension and those awaiting transplantation [53,55]. Interestingly, among a group of IPF patients with relatively mild disease (defined as FVC% predicted ≥ 80%), oxygen desaturation during the 6-MWT was the only significant predictor of death or progression within 12 months [56]. Little is known about the value of the 6MWT in earlier stages of the disease. However, the 6MWT is a validated test that can be easily performed outside of the hospital and requires few resources, making it a potential cost-effective screening tool.

### 2.11. Study Registration

The study was approved by the Medical Ethics Committee United (MEC-U, NL75303.100.20) and registered at https://clinicaltrials.gov/ct2/show/NCT05367349 (accessed on 20 December 2022); ID NCT 05367349.

### 2.12. Sample Size

The sample size was calculated based on our own retrospective data and literature. In pilot screening, we detected ILD changes in 13 of 73 (18%) asymptomatic, at-risk relatives, while in the literature, ILD changes were seen in 16–23% of the HRCT-scans [15,18,19,57]. In the present study, we will include 200 participants, with an expected ratio of 37 participants with the presence of ILD changes and 163 participants with the absence of ILD changes at HRCT scan.

### 2.13. Statistical Analysis

Categorical variables, including presence/absence of Velcro-like crackles, presence/absence of clubbing, MRC dyspnea scale, and *MUC5B* rs35705950 genotype will be expressed as numbers with percentages. Continuous variables, including FVC, TLC, DLCO (corrected for hemoglobin), VAS Cough score, MMP7 level, CCL18 level, KL-6 level, SP-D level, and 6MWT (distance in meters, sat O2 rest, sat O2 after 6MWT, sat O2 nadir, and sat O2 rest minus sat O2 nadir) will be displayed as means with standard deviations or medians with standard error, where appropriate.

The primary objective will be tested using a chi-square test or Fisher-exact test (as appropriate for categorical variables) and Student’s *t*-test or Mann–Whitney U test for continuous variables. Univariate logistic regression will be used to test which parameters differ between the groups. Parameters that are found to be diagnostic in univariate analysis with *p* < 0.20, will subsequently be assessed in a multivariate logistic regression analysis using the backward method to determine which are the strongest predictors for ILD changes on HRCT scan at baseline. In case too many parameters meet the *p* < 0.20 criterion, a cut-off of *p* < 0.10 will be used.

The secondary objective will be tested using the paired *t*-test, or Wilcoxon signed-rank test for the differences between the groups, and a repeated measure ANOVA test for the differences within the same group over time. After year 2, linear mixed models will be used to determine the strength of change of the parameters over time within and between both groups.

## 3. Discussion

The FLORIS study will be one of the first longitudinal cohort studies of this size to screen asymptomatic family members of FPF patients using an extensive screening protocol with tightly scheduled study visits. It will provide information for an evidence-based screening protocol for preclinical disease in at-risk relatives.

Description of study protocols in advance of obtaining study results is considered to add background information on the trial and reduce (publication) bias [58]. In the setting of FPF, the current study description may provide background for future screening protocols.

The primary aim of the current protocol is to determine the reliability of screening parameters to predict HRCT abnormalities. The HRCT scan has proven to be a valuable screening tool to detect interstitial changes in asymptomatic, at-risk relatives [15,17,18,19,20]. However, it is also expensive and not suitable for yearly use due to radiation exposure in healthy individuals. We hypothesize that in addition to currently used methods, a combination of patient-reported symptoms and physiological markers can serve as early predictors for the development of FPF. There will be an emphasis on easy-to-use, minimally invasive markers to investigate whether (a subset of) the screening activities could be transferred to the general practice in the future. Ideally, FLORIS will enable us to identify parameters that can accurately predict the presence of ILD changes on an HRCT scan, thereby making it possible to select who should undergo an HRCT scan and for whom this is not (yet) necessary. Conversely, parameters that lack predictive value for the presence of ILD changes on HRCT scan may be omitted from the clinical screening protocol.

With the exception of Rosas et al. [18], previous studies commonly set a minimum age limit to include only patients over 40 years of age [17,19,20], either over 40 years of age or within 10 years of age of diagnosis of youngest affected family members [15] or over 48 years [16]. Indeed, previous studies showed that age was significantly associated with the presence of interstitial changes on HRCT scan [5,15,17,19] and subjects with ILA on HRCT scan were older than subjects without ILA [15,19]. However, it is also known that surfactant-related gene mutations cause PF in relatively young subjects [8] and the phenomenon of genetic anticipation in telomere-related, gene mutation carriers. We chose not to set a minimum age, apart from a minimum of 18 years, in order to resemble our current clinical practice as closely as possible. In earlier studies, the screening population is heterogeneous, varying from family members of patients with sporadic or familial IPF [16], only familial IPF [18], or PF in at least two family members [19]. By only including first-degree, asymptomatic family members without a previous diagnosis of any ILD, we aim to include subjects predicted to have the highest chance of developing progressive fibrosis.

The benefit of the longitudinal set-up is twofold as it provides insight into (1) whether yearly screening, as is the current local protocol, is indeed necessary and (2) how clinical characteristics, predominantly pulmonary function, change in early disease. The latter is still largely unknown. A limitation of our study is that the follow-up time is relatively short at 2 years. Other studies have performed a second HRCT scan to assess radiologic progression after 4 [20] or 5 [15] years and Salisbury et al. showed that 63% of subjects with mild ILA progressed on HRCT scan after 5 years [15]. These studies showed that a significant proportion, though not all, of the subjects with ILD changes progressed over time. However, as the screening protocol in our clinic entails yearly screening, we chose to focus on the course of pulmonary function and other clinical parameters in this timespan. Since longitudinal follow-up of lung function was not described in the prior studies, FLORIS will provide new insight into the course of early FPF and yield information on the necessity of annual screening.

By performing a prospective study into the prognostic value of screening parameters in the target population and the appropriate clinical setting, the FLORIS study aims to further develop an evidence-based screening protocol for first-degree relatives of FPF patients. There will be an emphasis on easily operable parameters.

## Figures and Tables

**Table 1:** Study schedule of the FLORIS study.

	Study Period		
	Baseline	Year 1	Year 2
Screening and informed consent	X		
**Clinical parameters**			
Physical examination	X	X	X
HRCT scan	X		
Lung function tests	X	X	X
Blood tests (a)	X	X	X
Questionnaires (b)	X	X	X
**Exploratory parameters**			
Biomarkers (c)	X	X	X
6MWT	X	X	X
MUC5B rs35705950 genotype	X	X	X
Digital auscultation	X	X	X
Questionnaires (d)	X	X	X

(a) Complete blood count, kidney and liver function tests; (b) Medical research council (MRC) dyspnea scale, EuroQOL-5 dimensions (EQ-5D), fatigue-assessment scale (FAS), visual-analogue scale (VAS) for cough; (c) Krebs von den lungen 6 (KL6), surfactant protein-D (SP-D), chemokine (C-C motif) ligand 18 (CCL18) and matrix metalloproteinase 7 (MMP7); (d) Demographics and expositions, diseases in the family, risk perception, and reasons for participating. Definition of X = indicates that the action is performed or the parameter is measured at that time point.

## Data Availability

No data was presented in this manuscript.

## Abbreviations

ILD	interstitial lung disease
PF	pulmonary fibrosis
FPF	familial pulmonary fibrosis
IPF	idiopathic pulmonary fibrosis
RA	rheumatoid arthritis
HP	hypersensitivity pneumonitis
FVC	forced vital capacity
DLCO	diffusing capacity for carbon monoxide
UMC	Universitair Medisch Centrum
HRCT	high-resolution computed tomography
FLORIS study	Optimizing screening detection for early disease detection in FamiLial pulmOnaRy fibrosIS
MRC	Medical Research Council
EQ-5D	EuroQOL-5 Dimensions
FAS	fatigue assessment scale
VAS	visual analogue scale
KL6	Krebs von den lungen 6
SP-D	surfactant protein-D
CCL18	Chemokine (C-C motif) ligand 18
MMP7	matrix metalloproteinase 7
FILA	familial interstitial lung abnormalities
ILA	interstitial lung abnormalities
TLC	total lung capacity
6MWT	Six-minute walk test
Sat	saturation
